# Hypofractionated radiotherapy for breast cancer acceleration of the START A treatment regime: intermediate tolerance and efficacy

**DOI:** 10.1186/1748-717X-9-165

**Published:** 2014-07-24

**Authors:** Stefan Janssen, Christoph Glanzmann, Stephanie Lang, Sarah Verlaan, Tino Streller, Doris Wisler, Claudia Linsenmeier, Gabriela Studer

**Affiliations:** 1 Department of Radiation Oncology, University Hospital Zurich, Raemistrasse 100, CH-8091 Zurich, Switzerland

## Abstract

**Purpose:**

Prospective evaluation of accelerated hypofractionated radiotherapy (RT) in breast cancer patients treated with 41.6 Gy in 13 fractions plus boost delivered five times a week.

**Patients and methods:**

Between 03/2009 and 10/2012 98 consecutive patients aged >55 years presenting with breast cancer (invasive cancer: n = 95, ductal carcinoma in situ (DCIS): n = 3) after breast conserving surgery were treated in our institution with the following schedule: 41.6 Gy in 13 fractions 4 times a week and 9 or 12 Gy boost in 3 or 4 fractions (on day 5 each week), cumulative dose: 50.6 Gy in 3.2 weeks or 53.6 Gy in 3.4 weeks, respectively depending on resection status. 56 patients had a T1 tumor, 39 a T2 tumor. N-status was as follows: N0: n = 71, N1: n = 25, N2/3: n = 2. 23 patients (24%) received chemotherapy before RT. A prospectively planned follow-up (FU) visit with objective and subjective assessment of treatment tolerance (questionnaires) was performed 0 and 8 weeks after RT completion, and one, two and four years later, respectively.

**Results:**

Mean/median follow-up was 32/28 months (range: 12-56). After 2 years local control, loco-regional control and disease-free survival was 100%, 100%, and 98%, respectively. Overall survival was 96% at 2 years. Cosmetic outcome was very good with patients being satisfied or very satisfied in 99% (n = 86/87), 97% (n = 55/57) and 100% (n = 25/25) after one, two and four years after RT, respectively. No grade ≥ 2 pain was described in the 25 patients with a FU of at least 4 years. Fibrosis, telangiectasia and edema were found in 7-15%, 0-22% and 0-11% at one, two, and four years, respectively, and are comparable to other trials.

**Conclusion:**

The applied hypofractionated RT regime with single doses of 3.2 Gy plus boost doses of 9-12 Gy in 3–4 fractions applied in 5 sessions a week was effective and well tolerated on intermediate term FU.

## Introduction

Radiotherapy is the standard treatment after breast conserving surgery reducing the risk of local failure and improving overall survival in patients with breast cancer [1-4]. The most common fractionation schedule is 50 Gy in 25 fractions delivered over five weeks [5]. In the past years four large randomized hypofractionation trials with up to 10 years follow-up data showed equal results in terms of outcome and toxicity compared to the standard regime (Table 1) [6-10]. The Canadian trial and START B trial used a pragmatic regime of 42.5 Gy and 40.05 Gy in 16 or 15 fractions of 2.66 and 2.67 Gy, respectively. This was delivered five times a week adding up to about 3 weeks of therapy. In START A trial prescription dose of 39 Gy/41.6 Gy in 13 fractions 3×/week (single dose: 3/3.2 Gy) was applied in 5 weeks in order to keep the total treatment time comparable with the standard arm of 50 Gy in 5 weeks. The recurrence rate for the patients treated with 39 Gy in 13 fractions was slightly higher however not statistically significant compared to the control group. The experimental arms of START A and B have not been tested against each other in randomized trials. The START B regime holds the advantage of a short treatment time of 3 weeks.

**Table 1:** Literature review: randomized studies with different hypofractionation schedules in breast cancer patients, LR = local recurrence, CTx = chemotherapy, RT ln = periclavicular radiotherapy of lymph nodes, *not randomized

**Study**	**Pat.**	**FU (yrs)**	**Prescription dose in Gy (weeks)**	**LR**	**Induration**	**Teleangiectasia**	**Breast edema**	**Excellent or good cosmetic outcome**	**CTX**	**RT ln**	**Boost**
Whelan “Ontario Trial” (2010) [7]	1234	10	25×2 = 50	6,7%	10.4%			71.3%	11%	0%	0%
			16×2,66 = 42,5 (3)	6,2%	11.9%			69.8%			
Yarnold (2005)	1410	10	25×2 = 50	12,1%	28.6%	13.8%	12.6%		14%	21%	75%
Owen (2006) [9] “START Pilot study”			13×3,3 = 42,9 (5)		40.8%	14.3%	20.3%				(7×2 = 14 Gy)
			13×3,0 = 39 (5)	9,6%	20.4%	8.6%	10.8%				
				14,8%							
START A (2013) [8]	2236	10	25×2 = 50	6.7%	27.1%	7.2%	13.5%		35%	14%	61%
			13×3,2 = 41,6 (5)	5.6%	28.2%	7.1%	11.8%				(5×2 = 10 Gy)
			13×3,0 = 39 (5)	8.1%	21.6%	3.0%	7.3%				
START B (2013) [8]	2215	10	25×2 = 50	5.2%	17.4%	5.8%	9.0%		22%	7%	43%
			15×2,67 = 40 (3)	3.8%	14.3%	4.2%	5.1%				(5×2 = 10 Gy)
Kim (2013)* [12]	276	5	13×3 = 39 (3.2)	1.4%	3% (1y) 2% (2y)		20.4% (1y) 3% (2y)	83%	74%	0%	100% (3×3 = 9 Gy)
Present study*	98	3	13×3.2 = 41.6 (3.2)	0%	15% (1y) 7% (2y)	6% (1y) 22% (2y)	11% (1y) 0% (2y)	99% (1y) 97% (2y)	24%	3%	99% (3×3 = 9 Gy)

Here we present results of our prospective single institution experience treating breast cancer patients with an accelerated hypofractionated schedule with 41.6 Gy in 13 fractions 5 times a week and an additional boost in less than three and a half weeks.

## Methods

From 03/2009-10/2012 95 patients older than 55 years with invasive breast cancer and 3 patients with ductal carcinoma in situ (DCIS) were postoperatively treated in our institution with the following schedule:

For R0 resection: 41.6 Gy in 13 fractions 4 times a week and 9 Gy boost in 3 fractions (on day 5 each week), cumulative dose: 50.6 Gy in 3.2 weeks.

For R1 resection: 41.6 Gy in 13 fractions 4 times a week and 12 Gy boost in 4 fractions (on day 5 each week, afterwards daily), cumulative dose: 53.6 Gy in 3.4 weeks.

In the beginning of this RT regime in our institution in 2009 boost was carried out with 5-10×2 Gy (n = 10 included in this study) and three patients younger than 55 years were included (45 years: n = 2, 48 years: n = 1). In three patients an additional radiotherapy of periclavicular lymphatic drainage was carried out. 23 patients (24%) received chemotherapy before RT which in majority consisted of 4 courses of AC (adriamycin and cyclophosphamide) and 12 courses of taxol. Patient related parameters are summarized in Table 2.

**Table 2:** Treatment and patient related parameters

**Mean age (years)**	69 (range: 45-92)
**RT prescription dose**	13 × 3.2 = 41.6 Gy
**RT boost doses**	
no boost	1
1 × 3 = 3 Gy	1
3 × 3 = 9 Gy	65
4 × 3 = 12 Gy	23
5 × 2 = 10 Gy	2
8 × 2 = 16 Gy	2
10 × 2 = 20 Gy	4
**RT periclavicular lymphatics**	
**N-status**	3
N0	71
N1	25
N2	1
N3	1
**T-status**	
DCIS	3
T1	56
T2	39
T3	0
T4	0
**Grading**	
G1	16
G2	45
G3	33
Not known	4
R0	11
R1	87
**Hormone receptor**	
Positive	85
Negative	12
Not known	1
**Her2 status**	
Positive	10
Negative	86
Not known	2
**Chemotherapy**	23

Treatment planning was performed on CT datasets in Pinnacle treatment planning system using a 3-D conformal technique. All patients were treated in supine position using two to four tangential fields. Dose was prescribed to the PTV and was maintained between-5 and +7% of the reference dose according to the ICRU 50/62 report. In boost regions hot spots of >105% were avoided if possible.

In 90 patients boost was delivered with electrons (6-16 MeV). In 8 patients a photon boost was applied due to anatomical circumstances and larger volume. In recent patients RT concept was switched to 39.9 Gy in 15 fractions if a photon boost was indicated. In 30 patients (31%) a bolus material was used (0.5-1 cm) for treating the boost volume to optimize dose distribution at depth.

Exclusion criteria for this study were: Multicentric lesions or required boost volume exceeding one breast volume quadrant. In these cases a schedule with conventional fractionation was applied. Patients < 55 years were treated according to START B trial with 39.9 Gy in 15 fractions (reported elsewhere).

Acute toxicity was assessed weekly during treatment, at the end of therapy and 8 weeks later. Prospectively planned follow up (FU) visits with objective and subjective assessment of treatment tolerance (questionnaires) were performed one (n = 74), two (n = 57) and four years (n = 25) post radiation, respectively. Acute and late toxicity was assessed according to Common Terminology Criteria for Adverse Events (CTCAE) version 4.

### Statistical analysis

Survival analyses were performed using SPSS version 21. Ordinal and binary logistic regression analyses were carried out using commercially available Minitab software version 16.2.4.3. A p-value of less than 0.05 was considered as statistically significant.

## Results

### Disease control

Mean/median follow-up time was 32/28 months (range: 12-56). No local or loco-regional recurrence was observed. After 2 years local control, loco-regional control and disease-free survival was 100%, 100%, and 98%, respectively. Overall survival was 96%. At time of analysis 4 patients were dead after 25/30/42/52 months of FU: two of them due to distant metastases (meningiosis n = 1, disseminated metastases n = 1), two patients died from non-breast cancer related causes (pulmonary embolism n = 1, second primary in the lung with distant metastases n = 1).

### Early side effects

**Figure 1:**
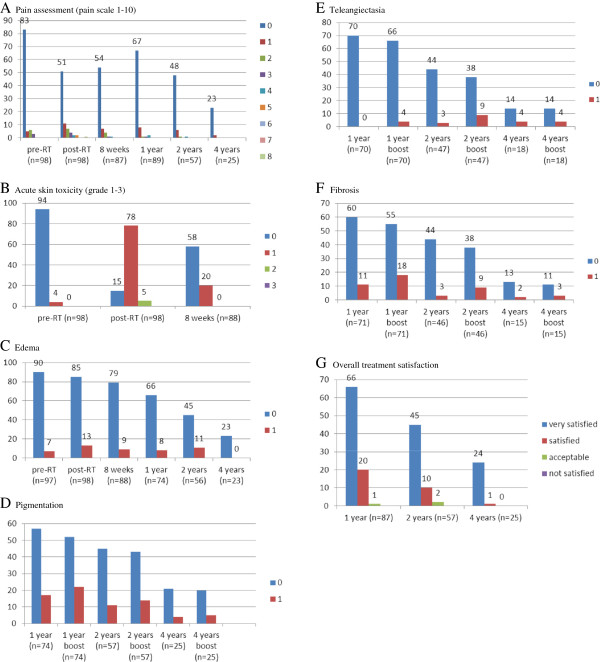
**A- G: Acute and intermediate toxicity and overall treatment satisfaction. A**: Pain assessment (pain scale 1-10). **B**: Acute skin toxicity. **C**: Edema. **D**: Pigmentation. **E**: Teleangiectasia. **F**: Fibrosis. **G**: Overall treatment satisfaction.

Grade 1 acute skin toxicity was observed in 80% (grade 2: 5%) directly after RT and in 23% after 8 weeks. Edema of the breast was seen in 7% before start of RT and in 13% and 10% immediately after RT completion and after 8 weeks, respectively. Pain within the treated breast was reported before RT start in 11% (grade 1: 5%, grade 2: 6%) and in 28% (grade 1: 11%, ≥ grade 2: 17%) and 15% (grade 1: 8%, ≥ grade 2: 7%) directly after RT and 8 weeks later, respectively (Figure 1A-C).

### Late term effects - objective late tolerance

Edema of the breast was observed in 11% (8/66), 20% (11/45) and 0% (0/23) after one, two and four years, respectively (Figure 1C). Hyperpigmentation of the whole breast (and boost region) was observed in 23% (30%), 19% (25%) and 16% (22%) after one, two and four years, respectively (Figure 1D). Telangiectasia of the irradiated breast (and boost region) was present in 0% (6%), 6% (19%) and 22% (22%), fibrosis in 15% (25%), 7% (20%) and 13% (20%) after one, two and four years, respectively (Figure 1E + F).

### Late term effects - subjective satisfaction

Patients were satisfied or very satisfied in 99%, 97% and 100% after one, two and four years, respectively (Figure 1G). One year after RT completion 13% (13/89) of the patients complained about pain (grade 1: 9%, ≥ grade 2: 4%). After two and four years pain was assessed in 15% (8/57) (grade 1: 11%, ≥ grade 2: 4%) and 8% (2/25), respectively (Figure 1A). No grade 2 pain or more was described after four years and no pain grade 5 or higher was observed in the entire FU period (except for three patients directly after RT completion).

### Predictive value of early symptoms on later tolerance

4/14 patients complaining about pain before RT start had persistence during further treatment. Pain status before and after RT/8 weeks later did not correlate with presence of pain in further FU period. Also edema of the breast immediately after RT did not correlate with its persistence during FU.

### Volumetric results

The mean volume of the breast was 682ccm (range: 258-1612). The mean volume of the boost region was 100ccm (range: 20-328). Additionally we assessed the volume of 105%, 110% and 115% isodoses outside the boost planning target volume (PTV). The mean volume of 43.7 Gy (105%), 45.8 Gy (110%), and 47.8 Gy (115%) isodose was 161ccm (range: 0-1349), 78ccm (range: 1-815), and 48ccm (range: 0-522), respectively.

No continuous correlation in logistic regression analyses was found in acute and late toxicity and dose-volume parameters like breast- and boost volume, boost dose or different isodose levels.

## Discussion

Our goal was to assess tumor control and treatment tolerance of accelerated hypofractionated RT with 3.2 Gy single doses and sequential margin-adapted hypofractionated boost in breast cancer patients. After a mean FU of almost three years patient were highly satisfied with cosmetic outcomes, the rate of late sequels was comparable to published data (Table 1) [6-9]. Limitations of our study are the limited number of patients and the relatively short FU. This also aggravates the logistic regression analyses after 4 years with a limited number of patients.

### Institutional development of hypofractionation schedules in breast cancer

Between 1967 and 1978 nearly all patients with post-mastectomy RT were treated with a hypofractionated regime (42.9 Gy in 10-13 fractions of 3.3 Gy 3×/week plus boost of 9.9 Gy/3 fractions), and a large part of patients with head and neck cancer received between 15 - 16 fractions of 3.3 Gy target dose, with a tolerance comparable to conventional fractionation. This regime has been abandoned after publication of a preliminary FU of a large fractionation trial [11] in favor of standard fractionation in a subgroup of 'glottic mobile'. Beginning in 1994, we used a hypofractionated regime in older patients (> 70 years) applying a whole breast dose of 45-48 Gy in 12-13 fractions of 3.0 Gy with excellent tolerance (unpublished results). After publication of the START trial in 2008, we decided to change our treatment regime in breast cancer based on the trial results in all patients.

### Late term tolerance

In hypofractionation late tissue tolerance is a major concern. We judged late reactions one, two and four years after RT completion using standardized questionnaires. In terms of fibrosis and edema our results were comparable to the four randomized trials. We found slightly more telangiectasia in the irradiated breast and boost region. This could be explained by the consistent use of boost in contrast to the above mentioned randomized trials where boost was applied in only 0-75% [6,7,9,10].

Patients judged cosmetic results as very satisfying or satisfying in 99%, 97% and 100% after one, two and four years, respectively. This compares favorably to the outcome of Kim et al. and Whelan et al. with 70% and 83%, respectively ([7,12] Table 1).

Patients with early pain/enhanced skin reaction before or directly after RT did not show to have any increased consecutive risk for pain or skin reactions in the future, however, the sample size at two and four years and the number of events is still too small to draw reliable conclusions from it.

### Early side effects

Acute side effects were assessed directly after RT and eight weeks later. After 8 weeks acute skin toxicity grade 1, edema of the breast and pain were present in 23%, 10%, and 15%, respectively. No grade 3 acute toxicity was observed. These results compare favorable to the results of Kim et al. assessing toxicity after RT at similar time points. The above mentioned randomized trials mainly focused on late tissue effects [6-9].

### Boost in hypofractionated schedules

The results of the START trials induced us to return to our formerly used hypofractionation regime in breast treatment. We changed our former regime to 5 fractions/week, as there is much evidence of increased efficacy while comparable tolerance applying such a dose regime with 5 instead of 3 fractions/week. Whole breast irradiation was carried out 4 times a week; additionally, we applied a margin-directed boost of 9 - 12 Gy in 3-4 fractions on day 5 of each week shown to be effective in conventional fractionation [13]. In contrast to the above mentioned randomized trials in which no boost [7] or a conventional fractionated boost of 5-7× 2.0 Gy = 10-14 Gy was applied in 43-75% of all cases [8,9], we applied a hypofractionated boost as described by Liau et al. and other study groups to further shorten overall treatment time [12,14-16]. Outcome was satisfactory with 100% local and loco-regional control and very satisfying or satisfying cosmesis in 100% at mean/median 32/38 months. These results stand in line with a recently published phase 2 trial showing 39 Gy in 13 fractions with a boost of 9 Gy in three fractions delivered in 3.2 weeks to be effective and tolerable [12].

In synopsis a boost of 9-12 Gy applied in our study to account for positive resection status seems to be safe and effective. This is a new aspect as the above mentioned randomized studies did not apply any boost or only in part of the cohort with single doses of 2.0 Gy. Lacking high level evidence the American Society for Radiation Oncology concluded that the boost indication is not clearly definable in hypofractionation of breast cancer [17]. However, boost RT is proven to have a benefit in conventional fractionated breast RT with an increase in local control from 89.8% to 92.8% [18]. Together with Kim et al. we showed a higher fractionated boost to be safe and effective in hypofractionated breast RT with no substantial compromise in cosmetic outcome. In contrast to a conventionally fractionated boost the benefit of shorter overall treatment time is still given.

Logistic regression analyses did not reveal any correlation of acute or late effects and isodose levels, PTVs of breast and boost or boost dose (9 Gy vs. 12 Gy). This may confirm our treatment approach with electron boost and two boost dose levels to be well tolerated after an intermediate FU time assessed in a limited cohort with few events (n individuals = 57 at 2 years and n = 25 at 4 years).

Further prospective data on accelerated hypofractionated breast RT especially in terms of boost regimes, application of chemotherapy and different tumor stages is needed.

## Conclusion

An accelerated START A regime of 41.6 Gy applying 4 fractions of 3.2 Gy/week plus a boost dose of 3-4 fractions of 3 Gy on day 5 results in a very satisfying tumor control and cosmesis after a median follow up of 28 months (range 12-56).

## Competing interests

There are no conflicts of interest to declare.

## Authors’ contribution

SJ drafted the text and tables, and created the Excel data set together with DW. CL/GS/CG designed the prospective evaluation forms/questionnaires. The dose volume analysis was performed by TS/SV/SL. CG and GS initiated and guided the project and reviewed of the manuscript. All authors read and approved the final manuscript.
